# Children’s Improvement of a Motor Response during Backward Falls through the Implementation of a Safe Fall Program

**DOI:** 10.3390/ijerph15122669

**Published:** 2018-11-27

**Authors:** Óscar DelCastillo-Andrés, Luis Toronjo-Hornillo, Manuel Rodríguez-López, Carolina Castañeda-Vázquez, María del Carmen Campos-Mesa

**Affiliations:** 1 Physical Education and Sports Department, University of Seville, 41013 Seville, Spain; lth00003@red.ujaen.es (L.T.-H.); carolinacv@us.es (C.C.-V.); mccampos@us.es (M.d.C.C.-M.); 2 Department of Research Methods in Education, University of Seville, 41013 Seville, Spain; rodri@us.es

**Keywords:** childhood injuries, falls, public health, education, health education

## Abstract

The World Health Organization has warned that, in children, the second cause of death from unintentional injuries are falls. The objective of this study was to analyze the motor response of primary schoolchildren when a backwards fall occurs. These analyses occurred before and after interventions of the Safe Fall program, which aims to teach safe and protected ways of backward falling. A quasi-experimental research design was used, with a sample of 122 Spanish (Sevillian) schoolchildren in the 10–12 age bracket. The INFOSECA ad-hoc observation scale was used for data collection: this scale registers 5 essential physical reactions throughout the process of a safe and protected backwards fall. After that, a number of descriptive, correlational and contrast statistics were applied. The value used in the McNemar test to establish statistical significance was *p* < 0.05. Results showed that over 85% of students had developed the competence to correctly perform all five physical motions that allow for a safer backward fall. The teaching of safe and protected techniques for falling backwards in child population in Primary Education is possible through the implementation of the Safe Fall program in Physical Education classes, which can help making falls safer, diminishing the risk and severity of the injuries they cause.

## 1. Introduction

Studying falls among schoolchildren has taken on considerable social importance, as it is a subject with close ties to public health, safety and child protection; for this reason, it has become a worldwide intervention priority in developed countries. The subject’s salience is also visible in numerous publications in high impact journals on education and public health [1,2,3,4,5].

The World Health Organization (WHO) has expressed concern about falls, as they are the second most common cause of children’s deaths resulting from unintentional injuries [6]. In Spain, different studies [7,8,9,10] have shown falls to be the third cause of child deaths from unintentional injuries. In Andalusia 4.3% of deaths caused from unintentional injuries derive from falls [8]. According to the Andalusian regional government, 26% of these deadly falls occur in the school setting [11,12]. In turn, in 2014, the Spanish Association of Pediatrics [13] pointed to falls as the leading cause of unintentional injuries in children under fourteen years of age. These statistics are similar at the international level, where the results obtained in Spain coincide with those of the European Alliance for Child Safety [14]. Regarding the type and frequency, several research projects have been carried out with the objective of determining what type of falls are the most common and how often they occur. These studies highlighted the frequency, severity, and the types of injuries that occur in the head, the upper and lower limbs, and the hip [7,10,13,15]. However, it can be observed that backwards falls, without being the most frequent, are responsible for the most serious types of injuries [9]. In support of the above statistics, the United States estimates the healthcare expenses arising from injuries from falls in children from 0 to 14 years to be $58 billion USD a year [16].

As awareness of this issue has grown, Spanish [8,9,13] and international [4,17,18] researchers have responded with prevention programs and research, with attempts to approach the issue from multiple sectors. However, prevention is not enough, as the risk of children falling while they practice sports or games is still present [5,19,20]. To provide a response to the problem associated to child falls beyond prevention programs, the current study proposes a proactive training program that serves as a new tool in the field of fall prevention. This program, Safe Fall [21], teaches children how to protect themselves when they fall. The program forms part of WHO proposals internationally, and in Spain, the Health Ministry [22] is contemplating multi-disciplinary programs aimed at minimizing the risk of injuries from falls. A recent study [23] showed that there is great demand for training in falls among future health and education professionals, owing to the current lack of training in this specific field.

Safe Fall has been designed to mitigate the risks posed by unintentional falls in school-age children. In turn, from a teaching viewpoint, it aims to improve the competences regarding this phenomenon of specialists in health, physical education, and sports. Safe Fall aims to contribute to existing fall prevention programs, giving education professionals an innovative and practical tool to proactively teach falling in a safe way.

The objective of this study is to analyze the motor response of primary schoolchildren when a backwards fall occurs before and after the implementation of the Safe Fall proactive program for the teaching of safe and protected ways of falling.

## 2. Materials and Methods

The pilot study is exploratory and quasi-experimental.

### 2.1. Sample

Convenience sampling was used for this research, resulting in a sample of 122 school-children. They were aged 10 (*n* = 44), 11 (*n* = 54) and 12 (*n* = 24) (36.1%, 44.3% and 19.7% respectively), and all attended state primary schools in Seville, Spain. We collected information about students in their 5th (*n* = 64) and 6th (*n* = 58) (52.8% and 47.2% respectively) year of primary education in the 2016/2017 academic year. Boys constituted 50.4% of the sample and girls 49.6%.

### 2.2. Procedure

The intervention of this study took place from 24 April to 2 June 2017, a six-week period. In this period, the Information Scale on Safe Ways of Falling (INFOSECA), ad-hoc observation scale (Appendix A), was used to collect empirical data.

These values allowed the students’ motor responses to be reliably and objectively collected. Likewise, the research complied with the approval of the Ethics Committee of the Biomedical Research of Andalusia and was focused on ensuring the safety of the child throughout the execution process. To guarantee physical safety, the test was performed on a polyurethane foam mat with a density of 20 kg/m^3^, covered with plasticized canvas and an air ejection system to absorb the energy generated by the fall. The teachers who implemented the program were completing a degree in primary education with the specialty of physical education and sports, and had been trained during the third and fourth year of their university degree within the subjects of Foundations of Physical Education, Physical Education Teaching, and Pre-Practicum Training. The data collection was carried out by professors of the University of Seville, members of the Physical Education, Health and Sport research team, and was directed by the main researcher of the project.

#### 2.2.1. Description of the Starting Position of the Test

The test is performed in a space where it is not possible to see the response to the pre-test of other classmates. The knees are flexed so the student is closer to the ground (a height measurer of 5 cm is placed), avoiding the accumulation of excessive inertia in the fall, that could entail some risk for the student due to the impact. The established height allows students to perform a motor response to the fall, assessed by the ungrouping of the hip or knees. From this position of grouping the lower limbs, the student carries their shoulders to the flexion with arms fully extended and palms down, which prevents the student from trying to hold to the researcher at the time of the fall. The grasp of the arms does not interfere with the motor response of the students since the response starts when the researcher releases the arms of the student. Finally, the student keeps the neck in a natural anatomical position, with closed eyes. The student closes his eyes to prevent him from taking reference points before the fall that could modify the motor response, such as looking at the feet causing neck flexion. The investigator holds the student by the wrists and causes a change of weight towards the heels of the subject. The exact moment is decided by the researcher by varying the time in which the student remains in imbalance or by grouping and ungrouping the student several times before releasing the grip. The first time the test is performed, without any training in falls, the fall is totally unexpected since the student does not know at any time what is going to happen and what will be evaluated. Regarding the post-test, the fall is more predictable because the student has performed the test before and has received training on safe ways of falling. The importance of the study is how the students respond to a fall before and after the Safe Fall program rather than the unexpectedness of the fall.

#### 2.2.2. Evaluation of the Test (Annex III)

A fall was defined as an event that results in a person coming to rest inadvertently on the ground or floor [6]. Using this definition, a tool has been developed that takes into account 5 fundamental reactions that take place throughout a backward, unexpected fall. Scoring is dichotomous and based on whether or not the 5 reactions are performed correctly. In relation to the neck variable, it is recorded whether the student responds to the fall by flexing or not flexing the neck. The hands variable evaluates whether or not the student puts the hands or elbows on the ground as a mechanism to stop the fall. The trunk variable evaluates if the student rolls on the back or falls on the back without control and therefore without reducing the force of the impact. Finally, the hip and knee variables are valued in an inverse manner, that is, if the initial grouping is maintained and, therefore, the height of the center of gravity does not increase before the fall occurs or, on the contrary, the student spreads instead of grouping (the authors believe that this data is valuable to understand the global structure of the fall). The test showed the following Kappa index values of reability: Neck variable, 0.93; Hands, 0.98; Trunk, 0.75; Hip, 0.82 and Knees variable, 0.84.

The proposal of these five elements (bending the neck, rolling on the back, not stopping the impact with the arms, bending the hips and knees), as elements involved in the increase in safety in the event of a fall, correspond to the data provided by different studies on the biomechanics of the fall [24], and conclude that landing strategies have a significant effect on the reduction of impact during a fall and could be effective in reducing the impact load that occurs in a fall. These authors conclude that landing strategies have a significant effect on reducing impact during a fall and might be effective to reduce the impact load of falling.

**Figure 1:**
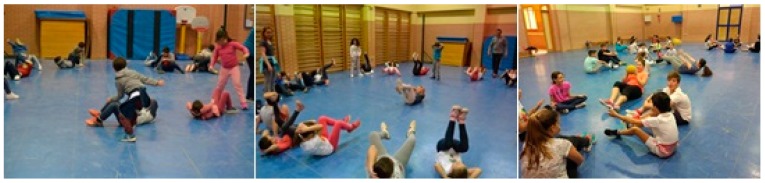
Implementation of safe fall program in primary school.

The program was implemented in four steps during Physical Education classes: a theory lesson on the incidence and effects of falls in school-aged children (1 h), pre-test, a lesson of practical Safe Fall skills, (50 min) and, finally, the application of specific Safe Fall exercises for ten minutes each day in the warm-up before the practical Physical Education session (10 practical sessions). These exercises are of increasing difficulty; teachers may choose to revise or change them based on how well students perform and progress in each session. (e.g., Figure 1). Upon completing the Safe Fall programme, in the last step the posttest data were collected to understand the changes in the motor response to unexpected backward falls.

We applied contrast statistics (chi-squared and bilateral exact significance) to find out whether there were significant differences because of the application of the Safe Fall program and not due to chance (Table 1). We analyzed the pairs of data from the pretest and posttest for each of the recorded direct variables by applying McNemar’s exact test. McNemar is a non-parametric test, for related samples, that allows us to contrast two measures of dichotomous categorical variables, which makes it a very suitable test for hypothesis contrasts for paired before/after type data. This test allows us to compare the change in the distribution of proportions between two measurements of a dichotomous variable and determine that the difference is not due to chance. Level of significance was established at *p* < 0.05. Data were analyzed using the SPSS statistical software (v.24.0, IBM, Armonk, NY, USA), applying descriptive, correlational and contrast statistics. 

All parents and guardians of participating minors were informed about the content and objectives of the program and gave informed consent for their children to participate in the research. 

## 3. Results

### 3.1. Backward-Fall in Primary Pupils

In the following we describe the five stages of protection for backward falls. Table 1 shows the results of the response obtained before and after the implementation. The results show that before receiving training in falls, less than one third bent the neck to protect the head from impact, and 99% of the children tried to stop the fall by supporting the upper extremities on the ground.

On the rolling up item, which is the gesture that when correctly performed allows for a timely change of the body surface hitting the ground [24], less than 10% of children performed this item correctly in the pre-test. Another notably low number was the 0.8% who correctly bent their hip. Finally, more than half the children maintained leg flexion, in accordance with the risk reduction parameters for backward falls.

### 3.2. Effects of the Program on the Competence of Pupils to Assimilate a Safe and Protected Backward Fall

As indicated in Table 1, there was an increase in the correct protection position in all the variables after the application of the program. In terms of neck flexion, the 76.4% of students who did not bend their neck in the pre-test fell to 9.8% post-intervention.

In the variable of not using the upper extremities to stop the fall, 98.4% of the students who did use them in the pre-test fell to 9.8% post-intervention. In the variable rolling up, in the pretest 91.9% performed this action incorrectly, and in the posttest 8.9% did it incorrectly. The hip flexion was incorrectly done by 98.4% before the program, and later only 13% did it incorrectly. Finally, knee flexion was incorrectly performed by 43.9% of the students before the program, reducing to 4.9%.

As indicated in Table 1, the change from pre- to post-intervention was significant at *p* < 0.05 for all five responses, indicating that the Safe Fall program produced changes in the students’ motor responses. An independent samples *t*-test indicated that the program was equally effective for both genders, as no significant differences in motor responses were recorded between boys and girls.

## 4. Discussion

The proposal to teach children safer falling techniques in Physical Education classes provides a new orientation for falls prevention and active protection aimed at the improvement of children safety and health [8,9,13,17,18]. It corresponds to the recommendations of the WHO [6], and the Spanish Ministry of Health [22], for the design of effective preventive programs. As these organizations point out, for these programs to be effective, they must be extensive and multi-disciplinary if the expected result is to eliminate the factors that cause the severity of the injuries when the fall does occur. In this sense, it is possible to teach school-age children protective motor responses to the most frequent responses that can lead to injuries during a fall [21,25].

The Safe Fall program is aimed at safeguarding minors when they fall by training motor responses associated with the protection of those areas of the body most at risk of injury in falls: head, upper limbs, hip or lower limbs [6,7,8,9,10]. Our results suggest that through repetition of the exercises performed in the Safe Fall program, students develop automaticity of the correct motor response, as has been shown in the elderly [26]. This would reduce the risk of injuries, as shown by the teaching of falls in judo [27]. 

The risk of falling increases in children between the ages of 6 and 12 because their development leads to participation in more complex games and activities that require greater dynamism [2,28]. Furthermore, in more developed societies, children in this age group are initiated into sporting activities, which in many cases entail a high risk of injury [8,10]. The improvement of basic motor skills proposed in the program, through the work of dynamic coordination, balance, and turns prepares minors to produce an adequate response to a fall, thus reducing risk of injury [21]. Essentially, the program aims to teach minors so that their motor response in the event of a fall is in accordance with parameters of greater protection and safety, and may contribute to a reduction in the risk of suffering injuries. After students have completed the Safe Fall program, repetition of the program’s exercises should be executed throughout all the academic years (ages 6–16) so that the response to a fall can be replicated. On this approach, it would be necessary to monitor the students trained in the program on safe falls and perform a new evaluation in order to obtain new data to establish the permanence or absence of the tested response. Likewise, it would be necessary to know what level of transfer can be expected in the event of a fall that occurs in a situation different from the context of the program. Furthermore, it would be important in order to establish a training program on falls, to understand the influence that the load in terms of volume and time of practice, have on the response obtained, so that the program continues to be effective in the event of an unexpected backward fall.

To correctly determine the methodology and progressions that allow effective responses to be established to facilitate the learning and automation of protective motor responses in backward falls, it is necessary to have prior knowledge of the circumstances and factors that take place during the fall [14,28]. The data obtained in this study is directly related to the possibility of reducing the injuries that occur most frequently in backward falls [10,15], such as, for example, decreasing the intensity of the impact of the head against the ground, and even avoiding the blow, providing protection against the most serious type of injury produced by this type of fall [9,24]. Although it has not been possible to compare this proposal with other programs designed to teach children to fall, the results obtained by other authors about a group of judokas between 16 and 30 years old, who train the falls in their sport, shows that in the event of an impending fall they suffer less injuries and less severe, compared to a group of people who did not practice professional sports [27].

This program directly influences one of the main causes of injuries among children [12,13,14]. Regarding this problem, a study carried out by the Andalusian government has determined that 26% of children’s falls occur in schools, which highlights the need to train primary school teachers in the specific area of falls and justifies that the program is a practical tool for teachers and those responsible for the education of school-age children [23]. Despite that, in the reviewed literature, no programs were found that teach school-age children to fall in a safe manner. 

In the comparison of the results obtained after this program between boys and girls, no significant differences have emerged in the data obtained, and it can be said that therefore, the program it is suitable for both genders despite the fact that boys are more likely to suffer falls [9,27].

Within the limitations of the study we must mention the scarce existing literature on programs to teach children to fall at school age, which prevents the comparison of data obtained with other studies. Another limiting aspect is the impossibility of correlating the motor response of the students, after receiving the training, in contexts different from those used in the laboratory conditions; although it should be noted that this limitation is determined by the predominant principle of protecting the physical integrity of the child. In successive research interventions it would be necessary to broaden the study of other forms of falling, such as sideways and forward. Finally, we want to point out in this paragraph the limitation of not establishing a control group to be able to contrast the consistency of our results. In future interventions, a control group will be established that, once the intervention has been carried out, will receive the same training that is carried out with the experimental group.

According to the results obtained, the study should be extended with the development of ways to fall forward and sideways, which contribute to the reduction of the risk of suffering injuries. This entails the need to develop new observation scales to evaluate the learning of protective gestures on falls occurring in those directions. Similarly, the sample could be extended to cover all state schools in Andalusia, Spain. Finally, the program could also be implemented in secondary schools, extending the age range from 12 to 16. In order to establish the possible positive correlation between the participation of the students in the program and the decrease of the number and severity of the injuries suffered as a consequence of a fall in everyday life, joint longitudinal studies and research in the area of Public Health would be needed, in which it could be established whether or not a decrease of the quantity or severity of injuries suffered as a consequence of a fall, as described by the WHO, is taking place in a target population that has received education regarding protected and safe falling techniques through the Safe Fall program. Likewise, new longitudinal studies must be carried out to determine the length of time required to implement the Safe Fall program, so that the motor responses considered adequate for safety in the event of a fall are automated by the students.

## 5. Conclusions

In conclusion, a Safe Fall training program implemented in Physical Education classes was successful in training appropriate motor responses to a fall in young children. Though not directly tested, the results suggest that students who participate in the Safe Fall program are at lesser risk of injury associated with a fall. This approach to preventing the harmful consequences of falls is a novel tool, which coincides with one of the needs raised by the WHO regarding the implementation of educational programmes based on falls research.

## Figures and Tables

**Table 1:** Statistics of assimilation of trained responses in falls backwards.

Variable	Pre-Test *N* (%)		Post-Test *N* (%)		X^2^	*p*
Bending the neck	28 (23.6)	94 (76.4)	110 (90.2) *	12 (9.8)	76.291	0.000
Not using hands	1 (1.6)	121 (98.4)	107 (90.2) *	15 (9.8)	104.009	0.000
Rolling up	9 (8.1)	113 (91.9)	111 (91.1) *	11 (8.9)	96.236	0.000
Bending hip	1 (1.6)	121 (98.4)	106 (87) *	16 (13)	103.010	0.000
Bending knees	68 (56.1)	54 (43.9)	116 (95.1) *	6 (4.9)	42.481	0.000

Note: * *p* < 0.05 in McNemar test; *N* = number of subjects; X^2^ = Chi-squared.

## Appendix A

**INFOSECA Scale for the Systematic Observation of: Backward Fall Student Assessment**

**School:**
**Number of subject on list:**
**Sex: Male/Female**
**Age:**
**Year:**
**Group:**
**Date:**
**Name of observer:**

**Description of starting position for activity assessment:**

1. Crouching position (buttocks slightly clear of the floor).

2. Wrists held by a classmate.

3. Position of imbalance with the trunk angled forwards while the classmate holds on to the wrists (they will later let go **without warning** to provoke the fall backwards).

When the classmate lets go of the wrists, the observer records on the following table whether the subject performed the actions in the different areas.

**INFOSECA scale for the systematic observation of: Backward Fall**

**Table:**

Criterion	Description	YES	NO
Neck	Bends the neck (chin down towards chest)		
Hands	Does not put hands on floor (protects the head)		
Trunk	Rolls into a ball and rolls over		
Hips	Keeps the hips bent		
Knees	Keeps the knees bent		

Minimum total assessment: 3 affirmative out of 5. Essential to have an affirmative response in “neck” item.
